# Elucidating the Effect of Step Cooling Heat Treatment on the Properties of 2.25 Cr–1.0 Mo Steel Welded with a Combination of GMAW Techniques Incorporating Metal-Cored Wires

**DOI:** 10.3390/ma14206033

**Published:** 2021-10-13

**Authors:** Subhash Das, Jay Vora, Vivek Patel, Joel Andersson, Danil Yurievich Pimenov, Khaled Giasin

**Affiliations:** 1 ITW India Private Limited, Vadodara 391775, India; Subhash.Das@millerwelds.com; 2 Department of Mechanical Engineering, School of Technology, Pandit Deendayal Energy University, Gandhinagar 382007, India; 3 Department of Engineering Science, University West, 46186 Trollhättan, Sweden; vivek.patel@hv.se (V.P.); joel.andersson@hv.se (J.A.); 4 Department of Automated Mechanical Engineering, South Ural State University, Lenin Prosp. 76, 454080 Chelyabinsk, Russia; danil_u@rambler.ru; 5 School of Mechanical and Design Engineering, University of Portsmouth, Portsmouth PO1 3DJ, UK; Khaled.giasin@port.ac.uk

**Keywords:** ductile-to-brittle transition temperatures (DBTT), gas metal arc welding process (GMAW), metal-cored, regulated metal deposition (RMD), step cooling heat treatment (SCHT), temper embrittlement, welding

## Abstract

The prospect of using metal-cored wires instead of solid wires during gas metal arc welding (GMAW) of 2.25 Cr–1.0 Mo steels embraces several challenges. The in-service requirements for the equipment made up of these steels are stringent. The major challenge faced by the manufacturers is temper embrittlement. In the current study, the temper embrittlement susceptibility of the welded joint was ascertained by subjecting it to step cooling heat treatment. A 25 mm thick 2.25 Cr–1.0 Mo weld joint was prepared using a combination of the regulated metal deposition (RMD) and GMAW processes incorporating metal-cored wires. After welding the plates were exposed to post-weld heat treatment followed by a rigorous step cooling heat treatment prescribed by API standards. The temper embrittlement susceptibility of the weld joint was ascertained by Bruscato X-factor as well as by formulating ductile-to-brittle transition temperature (DBTT) curves by carrying out the impact toughness testing at various temperatures. Detailed microscopy and hardness studies were also carried out. It was established from the study that the X-factor value for the welded joint was 15.4. The DBTT for the weld joint was found to occur at −37 °C which was well below 10 °C. Optical microscopy and scanning electron microscopy indicated the presence of carbides and the energy dispersive X-ray spectrometry studies indicated the presence of chromium and manganese-rich carbides along with the presence of sulfur near the grain boundaries. This study establishes a base for the usage of metal-cored wires particularly in high temperature and pressure application of Cr–Mo steels.

## 1. Introduction

2.25 Cr–1.0 Mo steels find their application in the fabrication of equipment used for processing under higher temperatures and pressures. This is because the mechanical and metallurgical properties of these steels ensure a safe working environment [1,2,3,4]. This equipment is subjected to severe varied cycles of temperature and pressure during their operation. The degradation/processes occurring at higher temperatures are different and more severe. Thus, the welding procedures, as well as corresponding heat treatment procedures, are simulated to meet required properties [5,6,7,8]. This equipment is all very large which makes arc welding techniques the obvious choice for fabrication. Cr–Mo steels possess good weldability for most processes, however, these alloys suffer from the temper embrittlement phenomenon when exposed to the temperature range of 370–550 °C over long periods [9]. There are several mechanisms for embrittlement out of which the two major phenomena reported by researchers are migration of impurity elements and changes in size and shape of carbides formed at the grain boundaries [10,11,12].

The factors responsible for temper embrittlement are chemical composition, temperature, holding time, and stress levels within the welded joint [6,13]. Thus it becomes necessary to assess the welded joints for their susceptibility to the temper embrittlement phenomenon. This phenomenon takes place after a longer duration of service and hence the assessment of its susceptibility is often time-consuming and tedious [14], as chemical composition plays an important role in temper embrittlement, the selection of welding filler metal also plays an important role [14]. Gas metal arc welding (GMAW) is one of the major processes used in fabrication incorporating solid wires [15,16,17]. However, fabricators face the challenge of productivity by using solid wires [18]. As an alternative, the solid wire can be replaced by tubular cored wires. These tubular wires enhance the current density and hence deposition rates increases. Two major types of cored wires are available, namely, flux-cored and metal-cored [19]. The flux-cored wires have a filling of shielding flux at the core which enhances the quality of welding due to efficient shielding but the deposition in kg is reduced as compared to solid wires. The welding process incorporating flux-cored wires is called flux-cored arc welding (FCAW) [20,21]. However, the metal-cored wires have filler metal at the core which enhances the current density as well as the deposition in kg which renders this process the most productive. This process is defined as metal cored arc welding (MCAW) [19].

Several attempts have been made to increase the productivity of this process by applying several techniques such as hybridization of processes, using cored wires instead of solid wires, and using variants of short-circuiting transfer. Several combinations of processes, such as GMAW–laser welding [22], plasma welding–GMAW [23], gas tungsten arc welding (GTAW)–laser welding [24], and plasma welding–laser welding [25], have been successfully demonstrated. For the ferrous steels in particular, several reports have been found wherein experimentation with various filler wire combinations for the root pass and fill-up passes have been carried out. In one of their studies Prajapati et al. [26] demonstrated that the weldment prepared by a combination of the GMAW–FCAW demonstrated the highest yield strength as well as toughness as compared to classical GMAW welds. However, it can be also concluded that the hybrid welds would have been taken more time as compared to the GMAW–FCAW joint as the deposition rates are lower in FCAW welding. In one of their other studies, the authors also demonstrated that the hybrid welds prepared by using flux-cored wires for root-pass and metal-cored wires for subsequent passes demonstrated higher properties than to other configurations under investigation [27]. Minimum angular distortion and maximum mechanical properties were reported for this combination. However, in another of their studies, the authors demonstrated the comparison between all three wires (flux-cored, metal-cored, and solid wires). It was reported that the metal-cored wires had the minimum angular distortion as compared to the other two. However, the toughness of metal-cored wires was inferior to the flux-cored wires [28]. This may be because the content of sulfur was slightly higher in metal-cored wires as compared to other consumables. Often the higher percentage of the impurity elements restricted the usage of metal-cored wires for Cr–Mo steels. However, in one of the studies by Das et al. [29] it was reported that the weldment prepared by depositing the root passes of the regulated metal deposition (RMD) process and filler passes with the GMAW process using metal-cored wires imparted excellent results. RMD is a modified short-circuit process favorable for root passes for mid-thickness plates (10–25 mm) [30,31,32,33]. From the summary above it is evident that the metal-cored wires enhance the productivity of the process and can be considered for welding of 2.25 Cr–1.0 Mo steels. However, to the best knowledge of the authors, the effect of using metal-cored wires with RMD and GMAW on temper embrittlement susceptibility of the weldment is yet to be reported in the open literature.

Two major methods for analyzing the temper embrittlement phenomenon are using a step cooling process and an isothermal aging process. The step cooling heat treatment (SCHT) method as designed by the American Petroleum Institute (API) [34] is a gradual cooling and holding of the weldment at specified temperatures and time. This typical cycle goes on non-stop for approximately 15 days and the weldment is subjected to the same followed by its testing. Compared to this, isothermal aging requires a longer period and is even more tedious and time-consuming. Hence, step cooling heat treatment is a widely used method for the assessment of temper embrittlement susceptibility [35,36,37]. However, the application of step-cooling in assessing the welded joints for temper embrittlement susceptibility is often attempted. Based on the summary above, it can be seen that any successful investigation in this direction would retain the use of metal-cored wires for manufacturing of high temperatures applications. This would also result in faster and more productive fabrication which will ultimately enhance the sustainability of the manufacturing ecosystem.

In the current work, an effort was made to study the result of step cooling heat treatment on the mechanical and metallurgical properties of the 2.25 Cr–1.0 Mo weldment. The root passes of the weldment were deposited by the RMD process and subsequent passes with the MCAW process with regime followed in the work reported by the authors. The temper embrittlement susceptibility of the weldment was analyzed by using the X-factor calculation and the ductile-to-brittle transition curves were prepared by doing a series of impact testing at various temperatures. Hardness evaluation and microscopic analysis were also carried out to understand the phenomenon occurring in the weldment. The authors firmly believe that the study will be of utmost importance to industries particularly engaged in the fabrication of equipment with high temperature and pressure applications.

## 2. Materials and Methods

**Figure 1:**
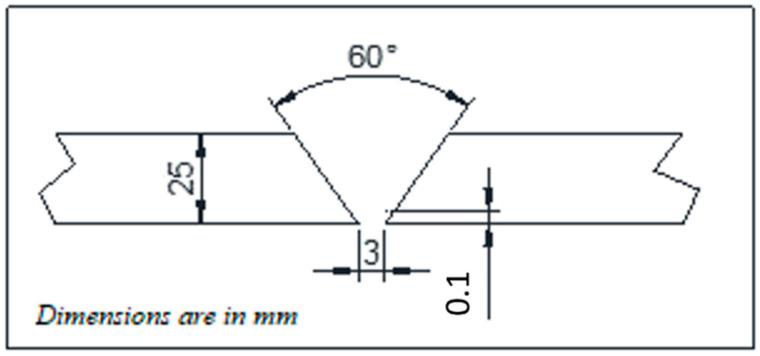
Weld configuration.

**Figure 2:**
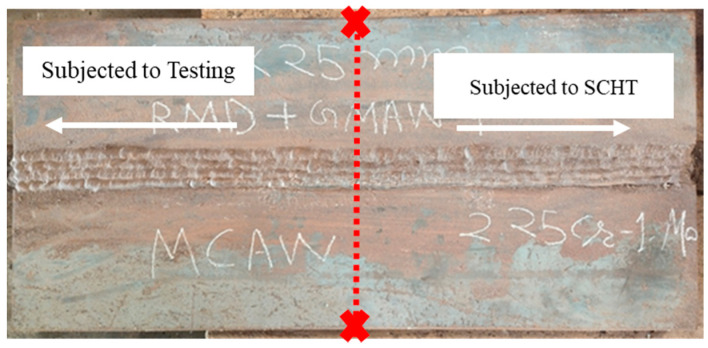
Welded plates.

2.25 Cr–1.0 Mo steel plates, also designated as SA 387 Gr 22 Cl 2, of 25 mm thickness were used for the current study. The chemical composition of the plate is shown in Table 1. The plates were wire-cut at the edges to form a 60° single V-groove and dimensions of 600 mm × 300 mm as shown in Figure 1. The plates were welded using metal-cored wire Megafil 237M (E90C-B3 H4) of 1.2 mm diameter with chemical composition as shown in Table 1. The welding was carried out using a Miller Continuum 500 machine capable of carrying out RMD as well as GMAW process as shown in Figure 1. The welding parameters were controlled as shown in Table 2. Root passes of the weld groove were carried out using the RMD process whereas subsequent passes were completed by the GMAW process incorporating metal-cored wires for both processes. The welded plates are as shown in Figure 2.

**Figure 3:**
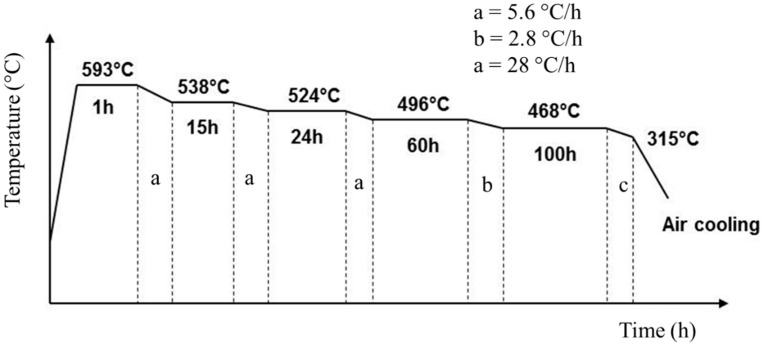
Step cooling heat treatment [14].

**Figure 4:**
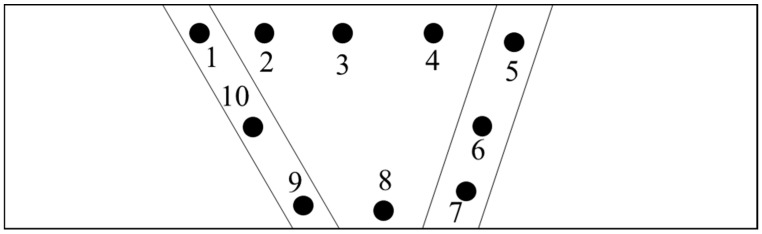
Hardness indentation across the weldment–SCHT condition.

After welding the plate was subjected to a post-weld heat treatment (PWHT) at 660 °C for 6 h at 100 °C per hour rate. The plate was cooled up to 400 °C within 4 h and then allowed to cool naturally. After PWHT the plate was cut into 2 halves and one half was subjected to step cooling heat treatment (SCHT) and the other to testing and characterization as shown in Figure 2. The step cooling heat treatment was carried out as shown in Figure 3. Thus one plate was subjected to PWHT and the other plate to SCHT (after PWHT) was available for further testing and characterization. The mechanical and metallurgical investigations were carried out for both heat-treated conditions as shown in Table 3. The macro- and microstructure analysis was carried out by extracting the sample from the center of the plates (both PWHT and SCHT conditions) in such a way that the entire weld area along with the heat-affected zone (HAZ) could be analyzed. The conventional sample preparation technique of mechanical grinding with different grades of emery powder (rough to fine) followed by polishing using alumina paste was followed. The samples were then etched using 2% Nital solution and observed under an Olympus inverted microscope with an image analyzer facility. A field emission scanning electron microscope (tabletop microscope, Hitachi) equipped with an energy-dispersive X-ray spectrometer (EDX) was utilized for a detailed microstructural study. Hardness variation across the weldment was carried out using a Bluestar Vickers hardness tester across different weld zones for SCHT conditions as shown in Figure 4. The indentations were made using a standard load of 10 kg for 10 s. Three indentations were taken at each location approximately 1 mm apart to ascertain the values at same location and average values were taken for analysis purposes.

**Figure 5:**
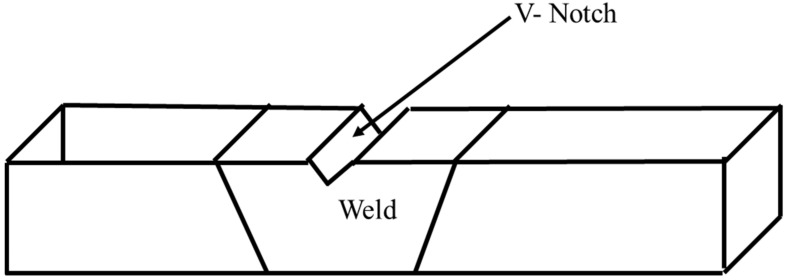
Location of V-notch in the impact testing specimen.

For analyzing the temper embrittlement susceptibility of the weldment after SCHT, chemical analysis was carried out using the spectroscopy technique at the center of the weld. More specifically the impurity content in ppm was analyzed and Bruscato’s X factor formula was applied. Additionally, Charpy V notch testing was carried out by preparing the samples according to the ASTM E23 standards from the center of the plate. The impact testing was carried out for both heat-treated conditions at different temperatures. At each temperature, three samples were tested and the average of these values was taken for further analysis. The V-notch was machined within the weld region so that the crack due to impact load developed in the weld zone only as shown in Figure 5. The impact specimen was removed from the plates of both the conditions (i.e., PWHT and SCHT). Impact testing was carried out at −80 °C, −60 °C, −40 °C, −29 °C, −18 °C, 0 °C, 10 °C, and 35° C. Ductile-to-brittle transition temperature (DBTT) curves were also plotted for both the conditions.

## 3. Results and Discussion

### 3.1. Macrostructure Analysis

**Figure 6:**
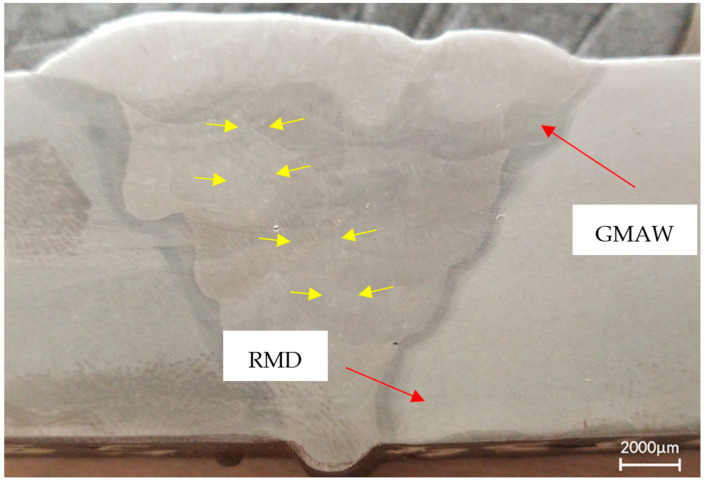
Macrostructure analysis of welded joint.

The macro image of the welded joint is shown in Figure 6. It can be observed that the weld deposited is completely free of defects such as lack of sidewall fusion (shown using small yellow arrows) or porosity. The root pass of the weld joint was deposited using RMD and subsequent passes using the GMAW technique. It can also be seen from the image that the bead placement and capping passes were deposited perfectly and the entire groove was filled with filler metal.

### 3.2. Examining Temper Embrittlement Susceptibility

The Bruscato X-factor is one of the efficient ways to predict the weld metal susceptibility to temper embrittlement. Several residual elements such as tin, phosphorus, antimony, and arsenic present in the filler metal as well as the base metal migrate to grain boundaries over time due to the exposure of these steels at a higher temperature for a prolonged time. This may cause a loss of toughness over some time. The X-factor is a numerical value calculated by the levels of residual elements in the weld deposit as per Equation (1) [14].

X = (10P + 5Sb + 4Sn + As)/100 (Content in ppm) (1)

The lesser value of these X-factor indicates the lower levels of tramp elements and ultimately better temper embrittlement resistance. The requirements of the X factor as per industry standards is 15. The chemical analysis of the 2.25 Cr–1.0 Mo weldment subjected to SCHT is as shown in Table 4. As per Equation (1), the value of the X-factor was calculated as 15.4. The value of the X-factor obtained can be deemed slightly on the higher side. This is because the welding was carried out with metal-cored wires. Due to constraints related to cost, the tramp elements in the metal-cored wires cannot be reduced below a certain limit and hence this increases the X-factor of the weldment subjected to SCHT. This is also one of the reasons for the limited use of metal-cored wires in welding of high temperature and pressure applications. To carry out further analysis impact testing was carried out at diverse temperatures and DBTT curves were drawn as we report in further sections.

### 3.3. Microstructural Analysis

**Figure 7:**
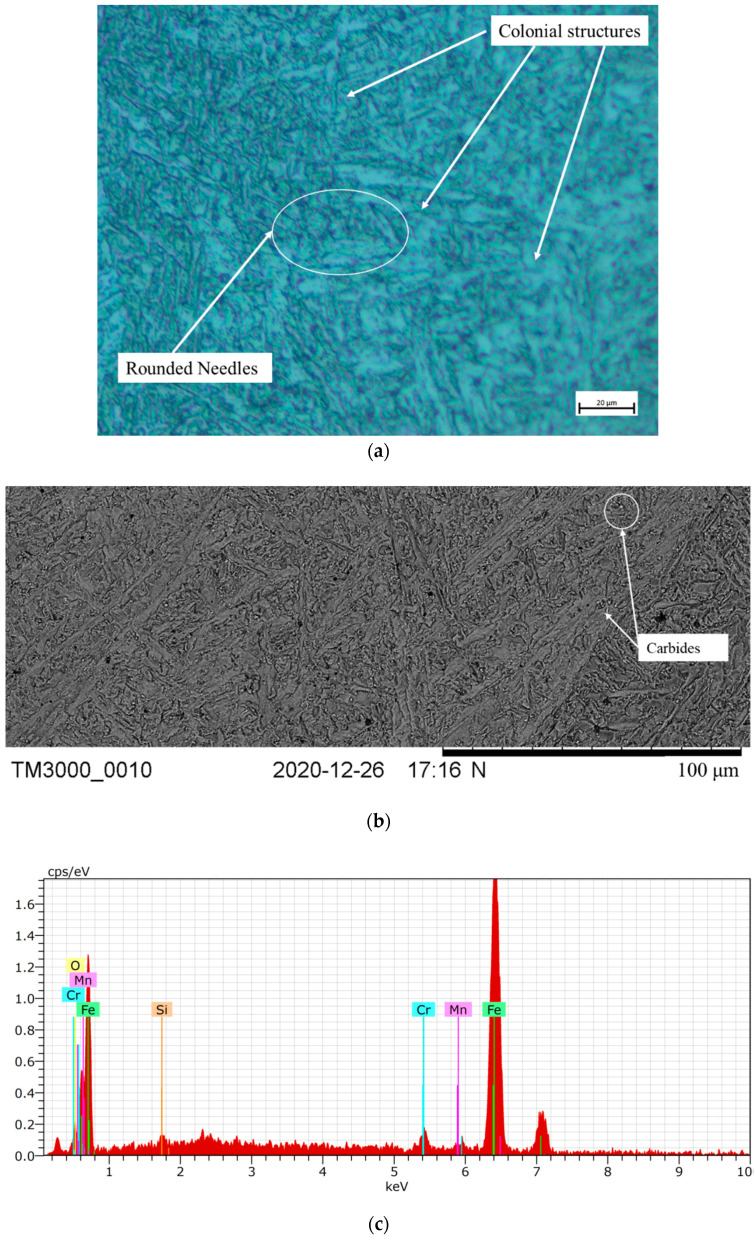
Microstructure analysis of the weld zone subjected to PWHT. (**a**) Optical microscopy, (**b**) SEM image, and (**c**) EDX analysis.

The weld micrographs for the samples subjected to PWHT and SCHT are as shown in Figure 7. The weld zone micrograph of the PWHT samples shows a colonial structure having blunt needle-shaped constituents (see Figure 7a). This is because 2.25 Cr–1.0 Mo steels are air-hardenable and hence they form hard martensitic structures on welding which are a mix of dendrites. On subjecting the weldment to PWHT, the diffusion of carbon and alloying elements takes place and the sharp needles are converted to blunt micro constituents on getting adequate time and temperature. Thus the structure can be considered as a mix of bainitic and tempered martensitic structures. During diffusion chromium- and molybdenum-rich carbides are also segregated at the grain boundaries as shown in Figure 7b. These carbides can be characterized by the bright spots along the grain boundaries. These are fine carbide precipitates segregated along the boundaries. The EDX results confirm that the carbides are rich in chromium and manganese (peaks) as shown in Figure 7c. These results are also in line with the fact that MX type and M23X type of carbides are reportedly precipitated [29,38].

**Figure 8:**
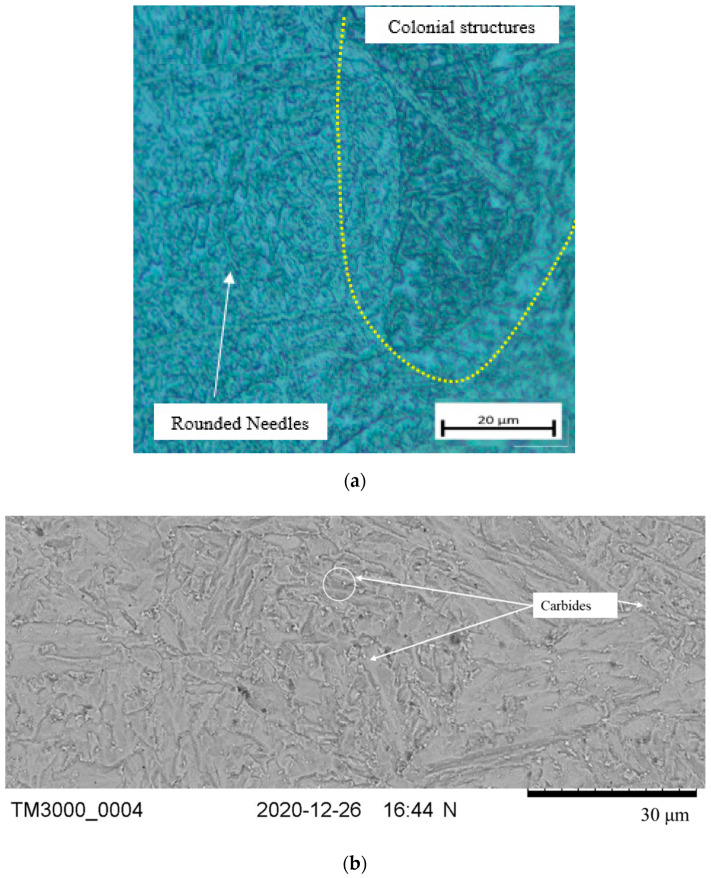

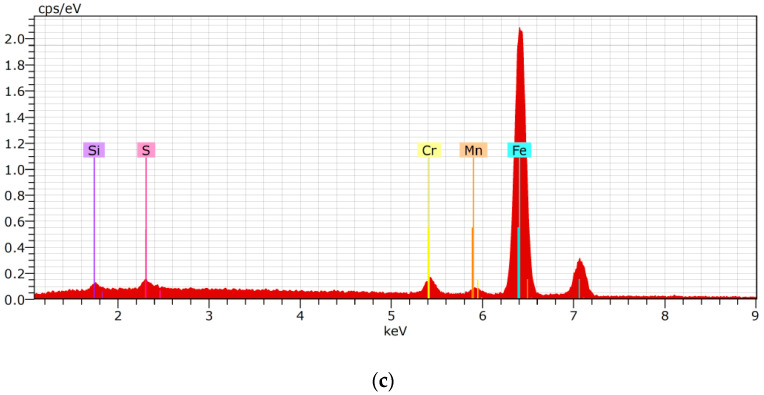
Microstructure analysis of the weld zone subjected to SCHT. (**a**) Optical microscopy, (**b**) SEM image, and (**c**) EDX analysis.

Similarly, micrographs of the weldment subjected to SCHT are shown in Figure 8a–c. It was observed from Figure 8a that nearly similar microstructures with rounded needles and carbides segregated at the grain boundaries were also observed in this case. The SEM image in Figure 8b indicates the occurrence of carbides (bright color particles) along the grain boundaries. However, the EDX results in Figure 8c indicate that apart from these carbides being rich in chromium and manganese, as indicated by the peaks, the presence of sulfur is also indicated. This is because due to SCHT the impurities move towards the grain boundaries near the carbides and segregate, which gives rise to temper embrittlement. However, apart from sulfur, no other impurity responsible for temper embrittlement was found near the grain boundaries. Thus it can be proposed that a single SCHT might have triggered the temper embrittlement phenomenon. The result was further was confirmed with the transition temperature studies in the next section.

### 3.4. Toughness and DBTT Values

**Figure 9:**
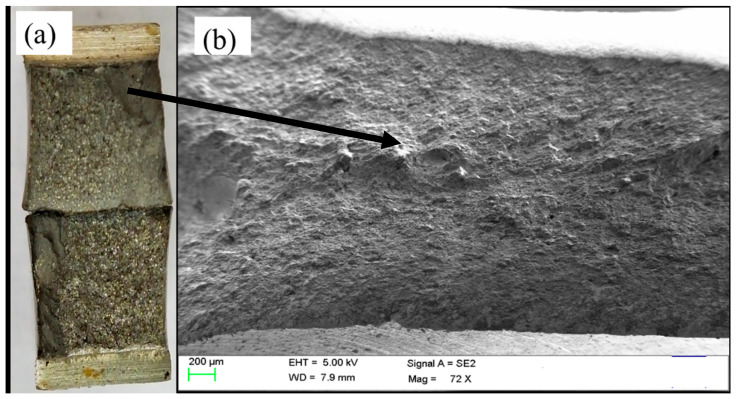
Fracture surface image of Charpy V-notch impact test results at −80 °C. (**a**) Fractured surface and (**b**) SEM image.

**Figure 10:**
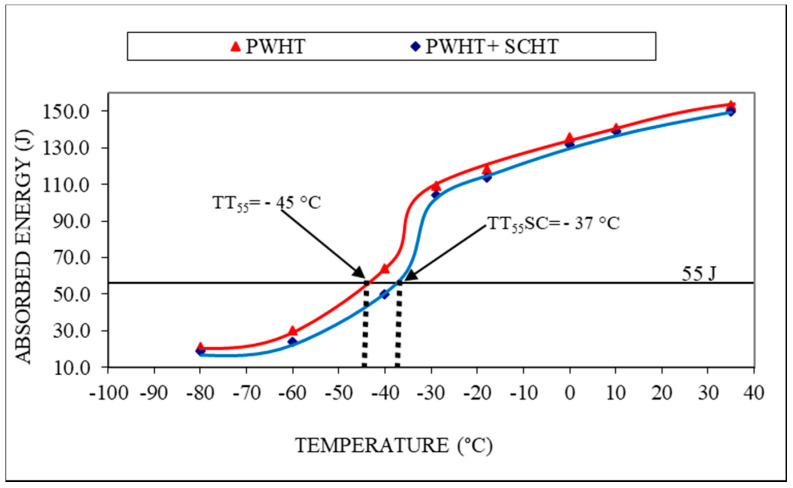
Impact toughness values and DBTT curves.

The impact toughness was ascertained with the help of the Charpy V-notch method, which was carried out for both the condition of weldment (i.e., PWHT and SCHT) at eight different temperatures. The samples were tested at the lowest temperature of −80 °C wherein for both the conditions the samples were broken in brittle mode with the least amount of energy absorbed as shown in Figure 9. From Figure 9a it can be seen that a flat surface on the fracture is seen which indicates the rupture of the specimen without any considerable stretching of yielding. This can be also characterized by the fact that, in SEM image Figure 9b wherein instead of stretched fibers, flat ridges are seen indicating limited yielding before fracture. This indicates the brittle mode of fracture. From Figure 10 it can be observed that the impact values for the weldment subjected only to PWHT were slightly better at all temperatures as compared to weldment subjected to SCHT. This is because 2.25 Cr–1.0 Mo steel tends to form carbides rich in alloying elements such as Cr and Mo. These carbides tend to impart toughness to the weldment by segregating at the grain boundaries. Due to this, when the impact load is given, they tend to restrict the failure and a higher amount of energy is absorbed before failure. At room temperature, the weldment shows a toughness of 153 J which is well above the intended values. In addition to this, the weldments, when subjected to prolonged heating during SCHT, tend to coarsen in size and shape due to the time available for diffusion. The number of carbides thus available at the grain boundaries reduces, and hence slightly lower impact properties are obtained for weldment subjected to SCHT.

To determine the susceptibility of the weldment for temper embrittlement, the ductile-to-brittle transition curves were sketched, as shown in Figure 10, such that they passed through the maximum points on the graph. It can be observed that the curve has shifted toward the right side which is for the weldment subjected to SCHT. The temperature at which the transition from ductile to brittle takes place is calculated by drawing a line at 55 J which is the requirement as per API 934A. This indicates the temperature to which the component can work safely without getting fractured due to embrittlement. The DBTT concept pertains to the fact that the weld microstructure of the equipment after years of operation (thermal cycles) undergoes a transition from the ductile mode to brittle mode. This is true with respect to a temperature after which there is a sharp fall in the ductility. Here, the DBTT value indicates the approximate temperature at which the equipment will not be able to have minimum required impact values (55 J) after undergoing SCHT. If DBTT values are lower than the working temperature of equipment, it is considered safe to use. Hence, lower temperatures of DBTT would indicate favorable results as equipment working temperature is generally above 0 °C [38].

It can be observed that the transition temperature value for weldment that has undergone PWHT (TT55) is −45 °C whereas the value for weldment with SCHT is (TT55SC) −37 °C. This result can be considered as a major outcome of the study as it proves the absence of any detrimental effects of embrittlement due to SCHT. In general, as per API 934 A, if the transition temperature if more than 10 °C the weldment is considered to be embrittled and not safe for work. Transition temperatures of −37 °C proved the limited effect of embrittlement of the weldment. This is also synchronous to the fact that as metal-cored wires were used for welding the value of impurities was on the higher side and an X factor value of 15.4 was obtained. The temper embrittlement occurred due to the presence of impurity elements such as phosphorus, antimony, arsenic, and tin present in the filler metal. These impurities tend to move towards the grain boundaries near the already-formed carbides and due to their segregation, the toughness of the region reduces drastically. However, in the present case, the segregation seemed to occur in a very sluggish manner and hence the absence of the subsequent amount of impurities near grain boundaries can be assumed as observed in the microanalysis in Figure 8. This supports the idea of using metal-cored wires for the high-temperature application components.

### 3.5. Hardness Variation

**Figure 11:**
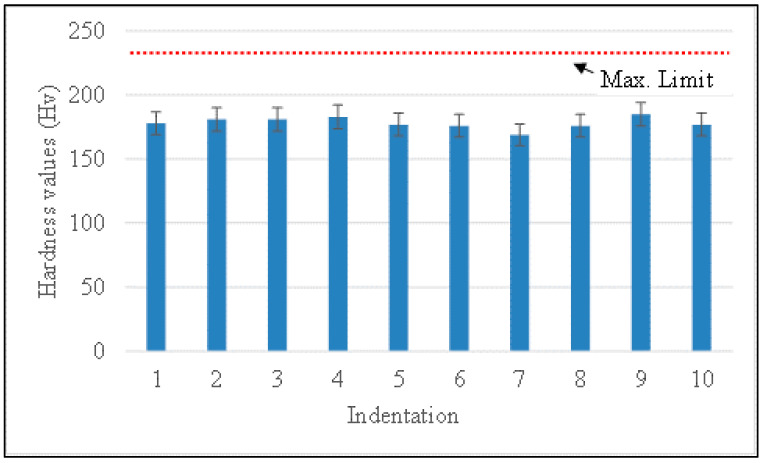
Hardness variations across the weldment.

One of the consequences of the embrittlement phenomenon is the fact that as brittleness increases the hardness also increases. As per API 934 A, the hardness number of 235 Hv is limited for the weldment. To reconfirm the absence of temper embrittlement phenomenon in the weldment after SCHT, the Vickers hardness survey was carried out as shown in Figure 4 across the weldment and results are shown in Figure 11. The indentations for microhardness were taken in the weld zone (including the HAZ) across the length and it can be observed that a maximum value of 183 Hv has been reported which is well below the maximum permitted value. This is because even after undergoing a step cooling heat treatment the 2.25 Cr–1.0 Mo weldment prepared using metal-cored wires has not shown detrimental effects due to carbide precipitation. This ensures a safe working environment for the components owing to lower temper embrittlement susceptibility. Additionally, the SCHT is always carried out in order to check the temper embrittlement susceptibility by the virtue of DBTT drawn with the help of impact testing results. After SCHT, yield strength is not considered as a major risk to the equipment and hence the study do not report the same. However, an approximation of the yield properties can be carried out following the methodology explained in the studies by Moshtaghi et al. [39,40].

## 4. Conclusions

This study attempted to investigate the susceptibility of the 2.25 Cr–1.0 Mo weldment prepared by using metal-cored wires using an arrangement of RMD and GMAW processes. With the help of detailed mechanical and metallurgical analysis, subsequent significant conclusions can be proposed:

- The weld macrostructures indicated a sound fusion without any evident defects such as lack of fusion or porosity. This established the weld parameters for the hybrid welding of 2.25 Cr–1.0 Mo using RMD and GMAW with metal-cored wires;
- Bruscato X-factor values of 15.4 indicated marginal chances of temper embrittlement. This was owing to the presence of a higher amount of impurity elements in the metal-cored wires;
- The weld microstructures showed evident colonial structures indicating a tempered martensitic structure with optimum hardness and requisite impact toughness;
- The impact toughness values of the weldment after PWHT and SCHT were found nearly similar, eliminating the chances of detrimental effects of SCHT;
- The EDX analysis indicated the presence of sulfur near the grain boundaries which might have been segregated due to SCHT. This also justifies the slightly higher X factor and it can be proposed from the study that the temper embrittlement phenomenon had might just be triggered due to a single SCHT;
- The DBTT curves indicted that the transition temperatures value was −37° C for the SCHT weldment which is well below the room temperature which hints at the safe operation of the equipment;
- The hardness variations across the weldment including HAZ were found to be fairly equal and their values were well below the acceptable values;
- The study introduces the prospect of using metal-cored wires for extreme service conditions.

## Figures and Tables

**Table 1:** Chemical composition of base metal and filler wire [29].

Content (% wt.)	Megafil 237M	Base metal 2.25 Cr–1.0 Mo
C	0.07	0.10
Mn	1.0	0.45
P	0.015	0.025
S	0.015	0.025
Si	0.3	0.50
Cr	2.3	2.25
Mo	1.1	1.00

**Table 2:** Welding parameters.

Parameter	RMD	GMAW
Current (A)	114–126	130–165
Voltage (V)	15–16	20–23
Feed speed of wire (in/min)	120	180–215
Welding Time (sec)	337	190–310
Travel speed (mm/min)	106	120–190
Heat input (kJ/mm)	1.05	1.18–1.60
Shielding gas	90% Ar–10% CO_2_	90% Ar–10% CO_2_
Gas flow rate (lpm)	18–20	18–20
Position	Manual (1G)	Manual (1G)

**Table 3:** Testing and characterization.

Testing	PWHT	SCHT
Macrostructure	✓	-------
Chemical analysis	-------	✓
Impact toughness	✓	✓
Microstructure	✓	✓
Hardness	-------	✓

**Table 4:** Chemical composition of weldment after SCHT.

Element	C	S	P	Mn	Si	Cr	Ni	Mo	Cu	V	Sb	Sn	Ar
Result (ppm)	730	130	130	9550	4960	23,600	180	10,100	990	120	20	30	20

## Data Availability

Data presented in this study is available in this article.

## Nomenclature

API	American Petroleum Institute
ASTM	American Society of Testing of Materials
Cr–Mo	Chrome–molybdenum steels
DBTT	Ductile-to-brittle transition temperature
EDX	Energy dispersive X-ray
FCAW	Flux-cored arc welding
GMAW	Gas metal arc welding
HAZ	Heat-affected zone
MCAW	Metal-cored arc welding
PWHT	Post-weld heat treatment
RMD	Regulated metal deposition
SCHT	Step cooling heat treatment
SEM	Scanning electron microscopy
SMAW	Shielded metal arc welding
